# Bioinformatics Analysis: The Regulatory Network of hsa_circ_0007843 and hsa_circ_0007331 in Colon Cancer

**DOI:** 10.1155/2021/6662897

**Published:** 2021-07-23

**Authors:** Zeping Han, Huafang Chen, Zhonghui Guo, Jianxia Zhu, Xingyi Xie, Yuguang Li, Jinhua He

**Affiliations:** ^1^Department of Laboratory Medicine, Central Hospital of Panyu District, Guangzhou, Guangdong 511400, China; ^2^Leizhou Center for Disease Control and Prevention, Leizhou, Guangdong 524200, China

## Abstract

**Objective:**

To analyze the molecular regulation network of circular RNA (circRNA) in colon cancer (CC) by bioinformatics method.

**Methods:**

hsa_circ_0007843 and hsa_circ_0007331 proved to be associated with CC in previous studies were chosen as the research object. ConSite database was used to predict the transcription factors associated with circRNA, and the CC-associated transcription factors were screened out after intersection. The CircInteractome database was used to predict the RNA-binding proteins (RBPs) interacting with circRNAs and screen out the CC-associated RBPs after an intersection. Furthermore, the CircInteractome database was used to predict the miRNAs interrelated with circRNAs, and the HMDD v3.2 database was used to search for miRNAs associated with CC. The target mRNAs of miRNA were predicted by the miRWalk v3.0 database. CC-associated target genes were screened out from the GeneCards database, and the upregulated genes were enriched and analyzed by the FunRich 3.1.3 software. Finally, the molecular regulatory network diagram of circRNA in CC was plotted.

**Results:**

The ConSite database predicted a total of 14 common transcription factors of hsa_circ_0007843 and hsa_circ_0007331, among which Snail, SOX17, HNF3, C-FOS, and ROR*α*-1 were related to CC. The CircInteractome database predicted that the RBPs interacting with these two circRNAs were AGO2 and EIF4A3, and both of them were related to CC. A total of 17 miRNAs interacting with hsa_circ_0007843 and hsa_circ_0007331 were predicted by CircInteractome database. miR-145-5p, miR-21, miR-330-5p, miR-326, and miR-766 were associated with CC according to the HMDDv3.2 database. miR-145-5p and miR-330-5p, lowly expressed in CC, were analyzed in the follow-up study. A total of 676 common target genes of these two miRNAs were predicted by the miRWalk3.0 database. And 57 target genes were involved in the occurrence and development of CC from the GeneCards database, with 23 genes downregulated and 34 genes upregulated. Additionally, GO analysis showed that the 34 upregulated genes were mainly enriched in biological processes such as signal transduction and cell communication. KEGG pathway analysis showed that the upregulated genes were closely related to integrin, ErbB receptor, and ALK1 signal pathways. Finally, a complete regulatory network of hsa_circ_0007843 and hsa_circ_0007331 in CC was proposed, whereby each one of the participants was either directly or indirectly associated and whose deregulation may result in CC progression.

**Conclusion:**

Predicting the molecular regulatory network of circRNAs by bioinformatics provides a new theoretical basis for further occurrence and development pathogenesis of CC and good guidance for future experimental research.

## 1. Background

Colon cancer (CC) is the most common lethal tumor of the digestive tract, which seriously threatens human life and health [1]. Its incidence rate is the third highest in females and the second highest in males. The clinical symptom of the patient with early CC is more hidden, and most patients are not discovered until the middle and late stages. Nowadays, surgery is still the first choice for the treatment of CC, supplemented by radiotherapy and chemotherapy, targeted therapy, and immunotherapy [2]. However, the problems of high recurrence rate and high metastasis rate after operation have not been effectively solved. The prognosis of patients with CC is still not very ideal, so it is necessary to find new treatments as soon as possible [3]. In recent years, a large number of studies have found that circular RNA (circRNA) plays an important role in the occurrence, development, metastasis, and invasion of CC [4–10] and can also be used as biomolecule markers for the diagnosis and prognosis [11]. circRNA is a new type of endogenous noncoding RNA, without a 5′-end cap and 3'-end poly (A) tail structure, showing a covalently closed loop [12]. Currently, it has been proven to act as sponges of microRNA (miRNA) or proteins, regulating the expression and alternative splicing of host genes, translating peptides, and participating in physiological and pathological processes of the body through these functions [13].

Our previous study found that circRNA was closely related to CC. hsa_circ_0007843 and hsa_circ_0007331 upregulated in CC were widely involved in the occurrence and development of CC, but the specific mechanism needed to be further elucidated [14, 15]. hsa_circ_0007843 is encoded by the ARHGAP32 gene (Rho GTPase-activating protein 32arhgap32), which locates at chr11:128993340-129034322 (http://www.circbase.org/). ARHGAP32 encodes a neuron-associated GTPase-activating protein that regulates dendritic spine morphology and strength by modulating Rho GTPase [16]. According to the literature, ARHGAP32 is widely involved in the occurrence and development of gastric cancer and liver cancer [17, 18], but its relationship with colon cancer has not been reported. hsa_circ_0007331 located at chr3:195101737-195112876 is encoded by the ACAP2 gene, a homolog of Caenorhabditis elegans CNT-1, which has a proapoptotic function and an identical phosphoinositide-binding pattern to that of CNT-1. It was reported that knockdown of ACAP2 blocks apoptosis in cancer cells in response to the chemotherapeutic antimetabolite 5-fluorouracil and that ACAP2 expression is downregulated in some esophageal cancers, leukemia, and lymphomas suggesting that ACAP2 inactivation or downregulation in human cells may contribute to cancer development [19]. However, the association of ACAP2 in colon cancer has not been reported.

**Figure 1:**
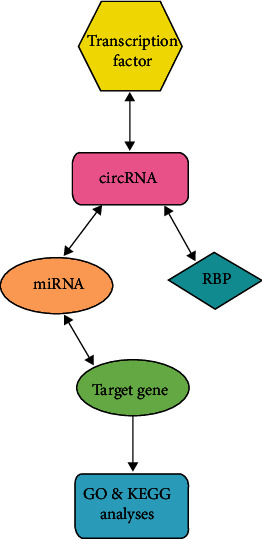
The schematic diagram of the research route.

Bioinformatics plays an important role in the field of life science. It mainly includes the generation, management, and analysis of multiple sets of high-throughput data in the field of biology and then comprehensively uses the theories and tools of mathematics, computer science, and life science to clarify the biological significance of these data [20]. Moreover, bioinformatics can be used to predict the regulatory network of circRNA in the occurrence and development of CC, which can better study the pathogenesis of CC and make the next research more targeted and clearer, so bioinformatics has become an indispensable research means in genome research. In this study, using bioinformatics methods, with hsa_circ_0007843 and hsa_circ_0007331 as the research object, we explore the regulatory relationship between it and upstream transcription factors, downstream RNA-binding proteins (RBPs), target miRNA, and miRNA target genes (Figure 1), to better understand the mechanism of circRNA in the occurrence, development, invasion, and metastasis of CC and provide new clues for the next experiment to verify its molecular regulatory network mechanism.

## 2. Materials and Methods

### 2.1. Identification of Transcription Factors Associated with circRNAs

ConSite database (http://consite.genereg.net/) is a web-based tool for predicting transcription factors by gene-binding sites, which can be used to find cisregulatory elements in gene sequences [21]. The transcription factors of hsa_circ_0007843 and hsa_circ_0007331 were predicted and analyzed by the ConSite database. The common transcription factors of the abovementioned circRNA were screened. The relationship between transcription factors and CC was queried by the PubMed database (http://www.ncbi.nlm.nih.gov/pubmed).

### 2.2. Prediction of RBPs Interacting with circRNAs

CircInteractome database (https://circinteractome.irp.nia.nih.gov/), for mapping RBP- and miRNA-binding sites on human circRNAs, searches public circRNA, miRNA, and RBP databases to provide bioinformatics analyses of binding sites on circRNAs and additionally analyzes miRNA and RBP sites on junction and junction-flanking sequences. Furthermore, it can also identify potential circRNAs that can act as RBP sponges, and design primers and siRNAs for circRNA [22]. The target circRNAs were submitted in the “circular RNA” of the CircInteractome database to obtain the RBPs that interact with circRNAs. Then, the common RBP of these two circular RNAs associated with CC was obtained through the PubMed database.

### 2.3. Prediction of miRNA Interacting with hsa_circ_0007843 and hsa_circ_0007331

Enter the prediction interface through “miRNA target sites,” and then, submit the target circRNAs to get the predicted miRNAs in the CircInteractome database.

### 2.4. Identification of CC-Associated miRNAs

The HMDD database is an experimentally confirmed database related to human miRNA diseases [23]. We searched for miRNAs that had been confirmed to be related to CC using HMDD v3.2 (http://www.cuilab.cn/hmdd/).

### 2.5. Target mRNA Prediction

miRWalk is an online software that covers the latest information about the interaction between mRNAs and miRNAs in humans, mice, dogs, and cattle. It can easily identify important miRNA targets to better understand the role of multiple miRNAs and optimize their genetic targets [24]. The target mRNAs of the above miRNAs are predicted by miRwalk v3.0 (http://mirwalk.umm.uni-heidelberg.de/), and the common target genes were screened after intersection.

### 2.6. Identification of CC-Related Target Genes

GeneCards database (https://www.genecards.org/) provides concise genome, proteome, transcriptome, disease, and functional data of all known and predicted human genes. It is a comprehensive database of human genes, which can query not only specific gene details but also all genes related to one disease [25]. All genes associated with CC can be obtained by simply typing “colon cancer” above “keywords.”

### 2.7. Functional Enrichment Analysis

FunRich is an open bioinformatics analysis system capable of functional enrichment and network analysis of genes and proteins [26]. GO and KEGG enrichment analysis of target genes can be performed by FunRich version 3.1.3 software (http://www.funrich.org/download).

### 2.8. Construction of the Regulatory Network of circRNAs in CC

Based on the genomic information above, the regulatory network diagram of circRNA in CC was constructed by Cytoscape software version 3.7.2 (http://www.cytoscape.org/index.html).

## 3. Results

### 3.1. Transcription Factors Associated with circRNAs

**Figure 2:**
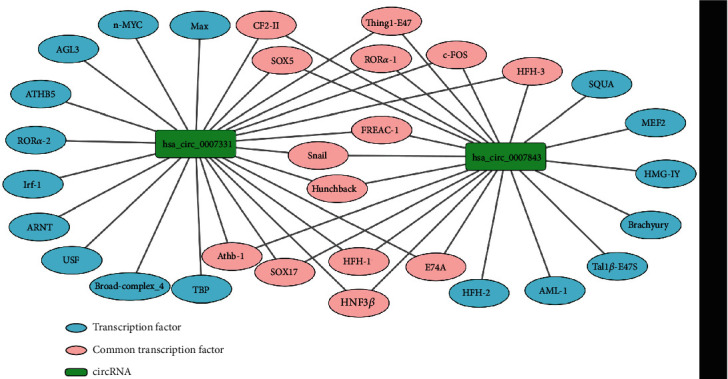
Transcription factors associated with circRNAs.

As transcription factors can act as upstream regulatory molecules of circRNA and affect its expression, we used the ConSite database to predict the upstream transcription factors of each circRNA. Under the condition of setting “cut-off” to 95%, 21 transcription factors of has_circ_0007843 and 25 transcription factors of has_circ_0007331 were predicted by the ConSite database. After intersection, there were 14 common transcription factors of the two circRNAs (Figure 2).

### 3.2. Transcription Factors Associated with CC

**Figure 3:**
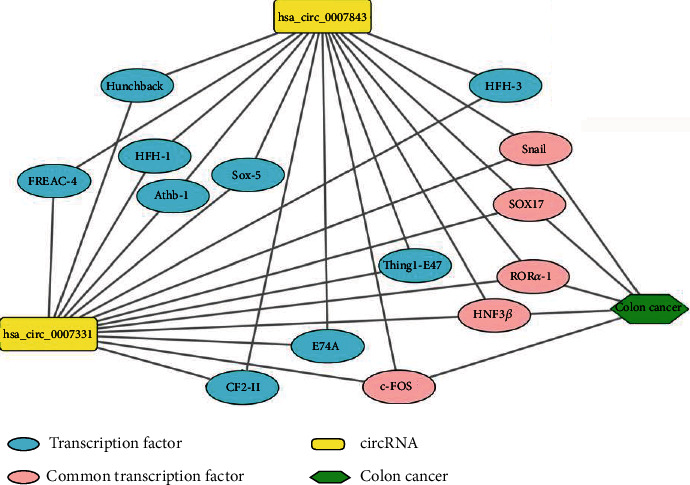
Transcription factors associated with colon cancer.

As shown in Figure 3, only 5 of the 14 transcription factors (Snail, SOX17, HNF3 *β*, c-FOS, and ROR*α*-1) are involved in the occurrence and development of colon cancer, consulting the literature through the PubMed database.

### 3.3. RBPs Interacting with circRNAs

**Figure 4:**
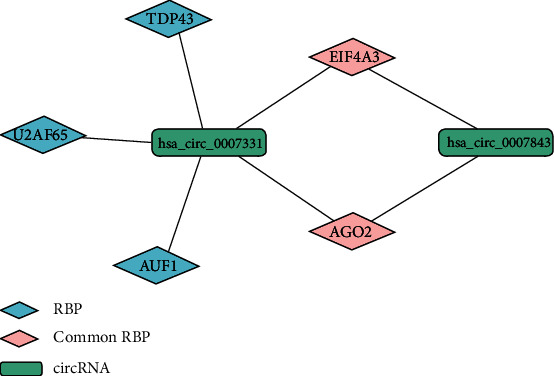
RBPs interacting with circRNAs.

After identifying the upstream regulatory transcription factors of circRNA, we continued to predict the downstream regulatory pathways of circRNA. The interaction with RBPs is also considered an important factor for investigating the function of circRNAs. The RBPs of has_circ_0007843 were EIF4A3 and AGO2, while those of has_circ_0007331 were AGO2, EIF4A3, AUF1, TDP43, and U2AF65 predicted by the CircInteractome database.  Figure 4 showed that EIF4A3 and AGO2 were the common RBPs of the two circRNAs, and both of them were associated with colon cancer [27, 28].

### 3.4. circRNA-Associated miRNAs

The CircInteractome database predicted that the miRNAs interacting with has_circ_0007843 were hsa-miR-1299, hsa-miR-326, hsa-miR-330-5p, hsa-miR-485-3p, hsa-miR-516a-5p, hsa-miR-543, hsa-miR-665, hsa-miR-766, hsa-miR-892b, and hsa-miR-921. Additionally, there were 7 miRNAs interacting with has_circ_0007331, such as hsa-miR-145-5p, hsa-miR-21, hsa-miR-590-5p, hsa-miR-433, hsa-miR-526b, hsa-miR-561, and hsa-miR-578.

### 3.5. CC-Associated miRNAs

A total of 139 miRNAs were confirmed to be associated with CC according to HMDD v3.2, which were intersected with the above 17 miRNAs to filter out five common miRNAs, including hsa-miR-326, hsa-miR-766, hsa-miR-330-5p, hsa-miR-145-5p, and hsa-miR-21. As known to all, acting as miRNA sponges, circRNAs can bind miRNAs through a miRNA response element (MRE) and negatively regulate their activity [29]. Since has_circ_0007843 and has_circ_0007331 were upregulated in CC, hsa-miR-145-5p and hsa-miR-330-5p, downregulated in CC, were selected as the objects of further research (Table 1).

### 3.6. Target mRNA Binding to miRNA

**Figure 5:**
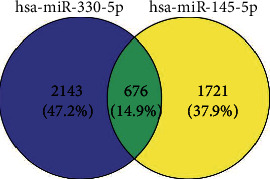
The common target mRNAs of hsa-miR-145-5p and hsa-miR-330-5p.

Click “miRNAs” from the main page of miRwalk v3.0, then enter hsa-miR-145-5p and hsa-miR-330-5p and predict the target mRNAs, respectively. Under the condition of setting the score to 1, a total of 2397and 2819 target mRNAs were identified for hsa-miR-145-5p and hsa-miR-330-5p, respectively. As shown in Figure 5, a total of 676 overlaps were identified for these two miRNAs.

### 3.7. The CC-Associated mRNAs

**Figure 6:**
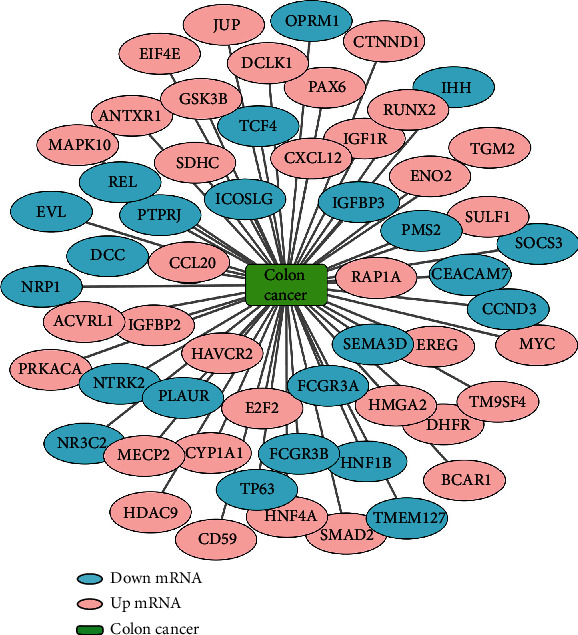
Intersection target genes associated with colon cancer.

1762 mRNAs associated with CC were obtained under the condition of “relevance” not less than 10 in the GeneCards database. A total of 57 mRNAs related to CC, with 34 mRNAs upregulated and 23 mRNAs downregulated, were obtained after taking an intersection with the 676 mRNAs above. As miRNAs are single-stranded RNA molecules that bind to targets in a base pair-mediated manner, resulting in the degradation or inhibition of the expression and function of target mRNAs, the expression of the target mRNAs was inverse to that of the miRNAs [30]. Then, 34 upregulated target mRNAs were included in the follow-up study (Figure 6).

### 3.8. GO and KEGG Enrichment Analysis

**Figure 7:**
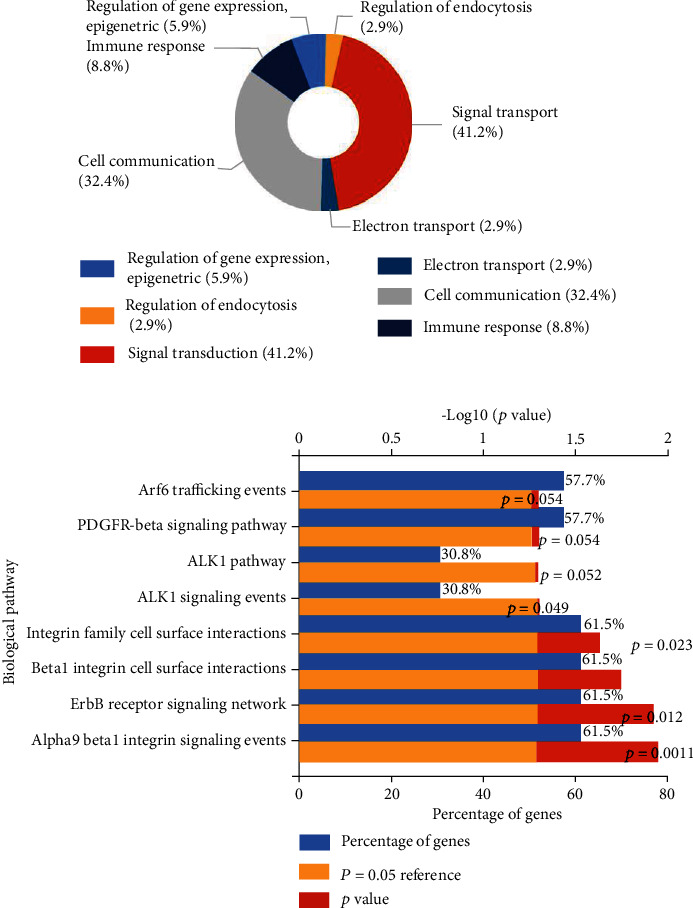
Enrichment analysis of target genes: (a) GO function enrichment; (b) KEGG pathway enrichment.

Enrichment analysis of the 34 upregulated target mRNAs was performed by FunRich 3.1.3. GO enrichment includes cellular components (CC), molecular functions (MF), and biological processes (BP). In biological processes, target genes were mainly concentrated in signal transduction and cell communication (Figure 7(a)). In KEGG pathway analysis, the most abundant pathways were *α*9*β*1 integrin signaling pathway, ErbB receptor signaling network, *β*1 integrin cell surface interaction, integrin family cell surface interaction, ALK1 signaling pathway, etc. (Figure 7(b)).

### 3.9. Functional Regulatory Network of circRNA in CC

**Figure 8:**
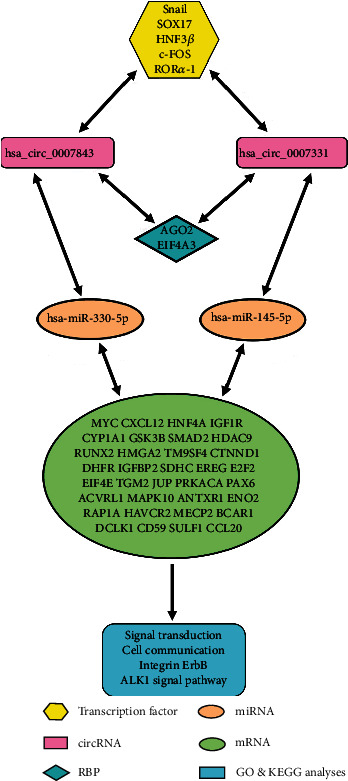
The regulatory network of circRNA in colon cancer.

Figure 8 presents the proposed regulatory network of circRNAs in CC.

## 4. Discussion

Bioinformatics is an interdisciplinary field of science that combines molecular biology, genetics, computer science, information engineering, mathematics, and statistics to solve data-intensive, large-scale biological problems from a computational perspective. It mainly focuses on modelling biological processes at the molecular level and making inferences from the data collected [31]. Moreover, bioinformatics can seamlessly store, mine, retrieve, and analyze data from genomics and proteomics using existing and emerging computing technologies and then construct computational models that speed up research and shorten the time of scientific research. The conclusions obtained from the analysis of the experimental data can be used to design the next stage of the experiment, and the computer can also be used to manage the experimental data and predict the structure and function of new genes and then to decode the complex biological information step by step, to provide a more clear direction for the diagnosis and treatment of various diseases.

In this study, we analyzed the molecular regulatory network of circRNA in the occurrence and development of CC by bioinformatics. hsa_circ_0007843 and hsa_circ_0007331, which were associated with CC in the previous study, were selected as the subjects of research. The expression of hsa_circ_0007843 and hsa_circ_0007331 in CC tissue was significantly higher than that in normal colon tissue. hsa_circ_0007843 acted as a miRNA sponge to regulate the expression of matrix metallopeptidase 2 (MMP2) by eliminating the inhibitory effect of miR-518c-5p on the translation of MMP2 gene, thus promoting the migration and invasion ability of SW480 cell [15]. And circRNA-ACAP2 (hsa_circ_0007331) could also affect the proliferation, migration, and invasion of CC SW480 cells by removing the inhibitory effect of miR-21-5p on Tiam1 expression [14].

As a competing endogenous RNA (ceRNA), circRNA can competitively bind to miRNA, thereby regulating the expression of its downstream target genes [32]. In this study, a total of 17 miRNAs interacting with hsa_circ_0007843 and hsa_circ_0007331 were predicted by the CircInteractome database. A total of 139 miRNAs associated with CC were found in HMDD v3.2, and the common miRNAs screened after taking the intersection with the above 17 miRNAs were hsa-miR-326, hsa-miR-766, hsa-miR-330-5p, hsa-miR-145-5p, and hsa-miR-21. Researches confirm that hsa-miR-326, hsa-miR-766, and hsa-miR-21 are upregulated in CC [33–36]. The expression of hsa-miR-330-5p in colon cancer tissue is significantly lower than that in adjacent tissues and negatively regulates the expression of integrin *α*5 (ITGA5), which affects the development of CC [37]. hsa-miR-145-5p, low-expressed in CC, targets CDCA3 to play a tumor suppressor role, acting as a biomarker for diagnosis and treatment [38]. It can also regulate RHBDD1 through EGFR-related signaling pathways to inhibit cell growth, invasion, migration, and tumorigenesis in colon cancer [39].

The miRwalk v3.0 online software predicted that 57 common target mRNAs of the two miRNAs (hsa-miR-330-5p and hsa-miR-145-5p) were closely associated with CC, with 23 downregulated and 34 upregulated according to literatures from the PubMed database. Enrichment analysis results showed that upregulated genes were mainly involved in related biological processes such as signal transduction and cell communication, while the KEGG pathway was mainly closely related to integrin, ErbB receptor, ALK1, and other signaling pathways.

It is well known that transcription factors are indispensable regulatory factors in gene expression. It was predicted by ConSite that there were 13 common transcription factors in hsa_circ_0007843 and hsa_circ_0007331. According to PubMed literature review, Snail, SOX17, HNF3*β*, c-FOS, and ROR*α*-1 have been confirmed to be associated with colon cancer. As a key transcription factor of epithelial-mesenchymal transformation (MET) in CC, Snail is upregulated in HT29 CC cells and regulates the expression of miRNA during EMT. Besides, studies have confirmed that the increased expression of Snail is significantly related to tumor size, lymph node metastasis, distant metastasis, clinical stage, and poor prognosis in CC patients [40, 41]. The SOX transcription factor family can regulate the classic Wnt signaling pathway, and when SOX17 is overexpressed in SW480 cells, it inhibits the activity of *β*-catenin/TCF in a dose-dependent manner, while also inhibiting cell proliferation [42]. HNF3*β*, an important transcription factor in gastrointestinal development, expressed in colorectal cancer, is lower than that in normal tissues. Its overexpression can inhibit the progression of CC through JAK-STAT3 signal transduction and serve as a potential target for antitumor therapy of CC [43]. The expression of c-FOS is associated with the metastasis and TNM stage of colon cancer. In the inflammatory microenvironment, GDF15 induces the invasion and metastasis of CC by activating c-FOS to regulate EMT gene [44]. Immunoassay revealed the expression of ROR*α*-1 protein in colon adenocarcinoma cells [45], but data on the regulatory relationship between ROR*α*-1 and CC have not been found.

circRNAs can combine with RBPs playing a crucial role in a variety of biological functions. The CircInteractome database predicted that the common RBPs of hsa_circ_0007843 and hsa_circ_0007331 were EIF4A3 and AGO2. In CC, EIF4A3 can combine with the long noncoding RNA H19, blocking the recruitment of EIF4A3 to cell cycle gene mRNA [21]. Studies by Huang et al. [23] have shown that AGO2 can be expressed in CC and may be related to the pathobiology of CC.

To synthesize the above results, the circRNA molecular regulatory network related to CC was constructed by bioinformatics analysis technology. Through this relatively systematic network, we found that the expression of hsa_circ_0007843, hsa_circ_0007331, and Snail in CC was upregulated, which promoted the growth and proliferation of CC cells; while the expression of hsa-miR-145-5p, hsa-miR-330-5p, and c-FOS was downregulated and played an inhibitory role. Based on this regulatory network, further research could provide more targeted treatment methods to reduce the expression of hsa_circ_0007843, hsa_circ_0007331, and Snail or increase the expression of hsa-miR-145-5p, hsa-miR-330-5p, and c-FOS to achieve the purpose of effective treatment of CC. The results of this study provided a valuable reference for elucidating the molecular mechanism of circRNA in the occurrence and development of CC and provided a theoretical basis for the screening of CC diagnostic markers and the selection of precise targets for drug treatment.

## Figures and Tables

**Table 1:** Colon cancer-associated miRNAs.

miRNA name	PMID	Description	Expression
hsa-miR-145	22766504	miR-145 regulates PAK4 via the MAPK pathway and exhibits an antitumor effect in human colon cells.	Downregulation
hsa-miR-330	22132977	Expression of miR-330 in various colon cancer cell lines varied fivefold between samples and correlated with in vitro gemcitabine resistance.	Downregulation
hsa-miR-326	22043014	The development of colonic inflammation in IL-10(-/-) mice was accompanied by upregulation in the expression of 10 miRNAs (miR-19a, miR-21, miR-31, etc.).	Upregulation
hsa-miR-766	30127618	Verified by qRT-PCR, the expression of hsa-miR-766 was upregulated in colon cancer cells.	Upregulation
hsa-miR-21	21279518	PDCD4 nuclear loss inversely correlates with miR-21 levels in colon carcinogenesis.	Upregulation

## Data Availability

The data that support results of the present study are available from CircInteractome database (https://circinteractome.irp.nia.nih.gov/), HMDD v3.2 (http://www.cuilab.cn/hmdd/), miRwalk v3.0 (http://mirwalk.umm.uni-heidelberg.de/), GeneCards database (https://www.genecards.org/), FunRich version 3.1.3 software (http://www.funrich.org/download), ConSite database (http://consite.genereg.net/), and PubMed (http://www.ncbi.nlm.nih.gov/pubmed).
